# Prevalence of directional asymmetry within the acetabulum and its implications for age estimation

**DOI:** 10.1007/s00414-025-03657-1

**Published:** 2025-12-06

**Authors:** Varsha Warrier, Marta San-Millán, Tanuj Kanchan

**Affiliations:** 1 School of Sciences, College of Science and Engineering, University of Derby, Derby, UK; 2 Medical Sciences Department, Clinical Anatomy, Embryology and Neuroscience Research Group (NEOMA), Faculty of Medicine, University of Girona, Girona, Spain; 3 Department of Forensic Medicine and Toxicology, All India Institute of Medical Sciences, Jodhpur, India

**Keywords:** Human identification, Forensic age estimation, Acetabulum, Directional asymmetry, Bayesian regression

## Abstract

Age estimation is a prerequisite for human identification. Within the skeletal framework, pelvic acetabular variables constitute a promising age marker. Previous investigations with the coxal bone have utilised either acetabulum for age estimation whilst assuming bilateral symmetry or selective side standardised practices, with two published studies reporting significant bilateral asymmetry within acetabular variables. The present study delves into this aspect of bilateral asymmetry further, and explores the prevalence, and impact of these side differences on age estimation. Data for analysis was obtained from 463 CT scans (195 females, 268 males) collected ethically from a medical institute in India previously. These scans were scored using a CT-based modification of the SanMillán-Rissech acetabular age estimation method, which utilises only the first five slightly modified variables of the original method as opposed to all seven. Collected data was then statistically analysed to illustrate the prevalence of asymmetry. The Wilcoxon test, Chi-square tests, mean % directional asymmetry values and equivalency ratios were utilised to assess population level lateralisation within the acetabulum. Furthermore, the association between asymmetry/ directionality, and biological sex and chronological age was investigated, and the impact of asymmetry on age estimation was evaluated using Bayesian regression analysis. Statistically significant bilateral differences were observed with the acetabular groove in females and the apex activity in males, and for all five variables the left acetabulum garnered older/ higher scores. Males largely demonstrated a greater degree of directionality wherein one side scored higher than the other more often, and patterns of directionality were seen to mostly increase with age in both sexes. The right, and/or younger scoring acetabulum consistently garnered most accurate age estimates, contradicting previous standardised practices of using the left acetabulum more, leading to its selective utilisation. Further, in-depth, investigation is wanting with regards to anatomical factors and lived experience of individuals capable of rationalizing these findings.

## Introduction

Age estimation constitutes one of the crucial parameters for human identification within forensic, medico-legal, humanitarian, and repatriation contexts [1–3]. Age estimation for investigative purposes is often undertaken through the analysis of ossification-fusion and/or surface changes transpiring within different bony elements of the skeletal framework [4–14]. Amongst these approaches, age estimation from the pelvis offers certain advantages. The human pelvis, in addition to housing multiple developmental and adult age markers which enable age estimation across a wider age cohort, is a robust and durable anatomical marker [15]. Within the pelvis, the hip bone—comprising the acetabulum and the auricular surface—undergoes physiological changes across a broad range of age cohorts [16]. Furthermore, the acetabulum and auricular surface are also known to demonstrate greater taphonomic resilience [17], when compared to other markers within the coxal bone. Thus, these markers confer several advantages to the age estimation process. Within the hip bone, the acetabulum is known to demonstrate higher accuracy for age estimation, when compared to other commonly utilised markers such as the pubic symphysis and the auricular surface [3, 13, 15, 18–23], rendering it a reliable marker for human identification.

Accurate and reliable age estimation is a prerequisite for positive identification and case resolution. An important factor which can, at times, impede accurate age estimation and human identification is asymmetry within the skeletal framework [2, 24, 25]. Prevalence of asymmetry within skeletal markers, if not accounted for during investigations, might often result in remains being misconstrued as belonging to different individuals, particularly in the case of fragmented remains. Significant bilateral differences in the appearance of skeletal markers, in turn, also impacts estimated age from paired elements within the skeleton and can compromise the accuracy of human identification [24]. Varying degrees of asymmetry within the skeletal structure, arising due to genetic predispositions, differential biomechanical loading and wear and tear, bipedal locomotion, and possibly, age and obstetrics, have been documented across literature [2, 26–29].

Studies on the prevalence of asymmetry within paired elements of the coxal bone, and its influence on age estimation have largely been limited to the pubic symphysis/pubic bone [2, 24, 29–31]. A standalone study, in 2024, undertook an investigation into the asymmetry associated with the acetabulum using acetabular articular cartilage surface areas for the assessment of acetabular fractures, and reported a difference of borderline significance [32]. There is, presently, a deficit of asymmetry-targeted investigations using surface changes of the acetabulum for age estimation, with most age estimation-targeted research having been undertaken with either acetabulum [12, 33–42]. A handful of investigations have examined both acetabula [19, 43–46], amongst which two investigations reported significant side differences [19, 46]. The other three studies utilised relatively smaller sample sizes, and/or different population groups, as compared to the present study, a plausible explanation for the discrepancies in findings between studies. Campanacho [46] within their thesis, utilised the left acetabulum for age estimation alone, despite the presence of significant side differences. Warrier et al. [19], on the other, undertook acetabular age estimation using both sides of the acetabulum. However, the extent and nature of this asymmetry was not analysed within this study. Asymmetry within the skeletal framework, by nature, can be majorly fluctuating or directional. Fluctuating asymmetry can be observed when skeletal asymmetry does not favour one side of the body over the other [26]. Directional asymmetry, on the other hand, is where one side consistently scores higher/appears older than its counterpart [26], and this can significantly impact the process of age estimation and human identification. Presence of directional asymmetry, in addition to hampering the identification of minimum number of individuals, also impacts age estimation by influencing the approach that should be utilised, i.e., consistently choosing one specific side over the other for age estimation, or giving equal weightage to both acetabula in order to get most accurate estimates of age. Furthermore, within previous publications, the association of asymmetry with chronological age and biological sex, and its implications for age estimation and human identification were not assessed. Given the higher accuracy and greater resilience associated with the acetabulum, as well as the relatively recent influx of acetabulum-targeted research for human identification [13, 16, 18, 19, 39, 41, 42, 44, 47–53], asymmetry within this marker and its consequences for age estimation warrant further scrutiny.

The present study aims to investigate the prevalence of asymmetry between the right and left acetabula, and its potential implications for forensic age estimation. Furthermore, the study attempts to establish patterns, if any, between the incidence of asymmetry, and chronological age and biological sex of the population.

## Materials and methods

### Sample

The samples employed within the present research were collected and analysed by the primary author between 2019 and 2023 at the Department of Forensic Medicine and Toxicology, All India Institute of Medical Sciences (AIIMS), Jodhpur, India. Ethical approval for this study was obtained from the Institutional Ethics Committee (AIIMS/IEC/2019–20/1007) of the All India Institute of Medical Sciences (AIIMS), Jodhpur, India. Computed tomographic images from participants aged 10 years and above, scheduled to undergo routine pelvic/abdomen radiological investigations for diagnostic purposes at the Department of Diagnostic and Interventional Radiology, AIIMS, Jodhpur were incorporated into the study. Such individuals/their guardians were approached and informed about the scope and aims of study, and informed consent was obtained prior to incorporating their CT images into the study. CT image of each consenting patient was collected following proof of age verification through valid government issued documents. Individuals who could not produce any valid documentation for proof of age, as well as those presenting with skeletal injuries/bony deformities within the acetabular region, capable of impacting the analysis of interest, were excluded from the study. Given the nature of this investigation, CT images of individuals, where either acetabulum was not examinable due to fractures, or, imaging and technical errors, were excluded from the analysis. Additionally, only mature acetabula with a completely obliterated triradiate cartilage were included for analysis, to allow for age estimation to be undertaken [41].

The final study sample comprised of CT images obtained from 463 consenting individuals (195 females with a mean age ± SD of 49.23 ± 15.762 years; 268 males with a mean age ± SD of 46.54 ± 18.009 years), ranging in age from 10 to 88 years, with an overall mean age ± SD of 47.67 ± 17.132 years. Age and sex-wise distribution of this study sample has been tabulated in Table 1. Although the complete fusion of three elements of the os coxa are reported to transpire around 15–16 years within an Indian population [54], five individuals between ages 10 and 14 years displayed a completely obliterated triradiate cartilage, and were thus, included for analysis. The inclusion of such outliers was not considered problematic here, as the study is directed towards evaluating the impact of asymmetry, rather than deriving specific age estimation models.

**Table 1:** Age-wise sex distribution of the study sample (*N* = 463)

Age range	F (%)	M (%)	*N* (%)
10–19	08 (4.10)	15 (5.60)	23 (4.97)
20–29	19 (9.74)	47 (17.54)	66 (14.25)
30–39	20 (10.26)	34 (12.70)	54 (11.66)
40–49	41 (21.03)	44 (16.41)	85 (18.36)
50–59	50 (25.64)	47 (17.54)	97 (20.95)
60–69	37 (18.97)	49 (18.28)	86 (18.57)
70–79	18 (9.23)	26 (9.70)	44 (9.51)
80–88	02 (1.03)	06 (2.23)	08 (1.73)
Total	195 (100.00)	268 (100.00)	463 (100.00)

*F* Number of females, *M * number of males, *N * Total number of participants, *%* corresponding proportion of individuals in each age class

*Percentage values have been rounded off to two decimal places

For the purpose of data analysis within this study, this sample was divided into three broad age classes as shown in Table 2. These age classes are based on Calce’s study from 2012 [48] wherein individuals were grouped as young (17–39 years), middle (40–64 years), and old (≥ 65 years), with the young age class modified slightly to include the 10–16 year olds of this study sample (effectively creating young as 10–39 years). Owing to the approach of sample collection [19], i.e., CT scans obtained from consenting individuals undergoing radiographic examinations for interventional and diagnostic purposes, a homogenous study set could not be ensured, with a greater preponderance of 40–64 year olds, and a relatively lower representation of younger and older age classes. For the same reason, an equal representation of biological males and females could not be ensured.

**Table 2:** Age-wise sex distribution of the study sample according to Calce’s age classes

Age range	F (%)	M (%)	*N* (%)
10–39 years	47 (24.10)	96 (35.80)	143 (30.90)
40–64 years	110 (56.40)	91 (34.00)	201 (43.40)
65 + years	38 (19.50)	81 (30.20)	119 (25.70)
Total	195 (100.00)	268 (100.00)	463 (100.00)

*F * Number of females, *M * number of males, *N * Total number of participants, *% *corresponding proportion of individuals in each age class

*Percentage values have been rounded off to two decimal places

### Scoring method

CT images utilised within the present study were primarily assigned unique codes to blind the observer(s) to any identifying parameters. Each CT image was individually processed, and each half of the pelvis was examined separately, and at different times, to circumvent bias. Thus, a total of 926 acetabula (463 paired acetabula) were examined and scored using the ordinal aging method described within Warrier et al., [19] which is a CT-based modification of the SanMillán-Rissech method proposed in 2017 [41]. This modified method utilised only the first five acetabular variables described within the original study, i.e., acetabular groove, acetabular rim shape, acetabular rim porosity, apex activity, and activity on the outer edge of the acetabular fossa. The remaining two variables of texture and bone density in the centre of the acetabular fossa and activity in the acetabular fossa could not be visualized with sufficient clarity as all CT images appeared to display the ivory shine, which is characteristic to young individuals alone, and morphological features of coarse granularity and bone chords could not be appreciated on CT images. Thus, these two features were excluded from the analysis to avoid skewing of results. However, this did not appear to impact the overall accuracy associated with the method [19, 41]. Ordinal scores assigned to paired acetabula for the entire study sample, using this modified method, were subsequently evaluated for the prevalence of side differences, if any, using different statistical computations.

### Statistical analysis

Initially, a Kolmogorov-Smirnov test was employed to assess whether the variables followed a normal distribution. If normality was not met, non-parametric statistical tests were applied. A p-value < 0.001 was obtained, illustrating a non-normal distribution for all five ordinally scored variables for both, the right and left acetabula. Thus, non-parametric equivalents of all tests were employed beyond this point.

#### Prevalence of directional asymmetry

Sex differences in the scoring of each of the five acetabular variables were evaluated using the Mann-Whitney U test. Subsequently, the Wilcoxon signed-rank test was applied to assess bilateral differences in the scoring of the acetabular variables, separately for each biological sex. Previous research using a similar sample has indicated the presence of asymmetry within acetabular features [19]. Under the assumption that a corroborative finding will be obtained within the present study (which was validated by statistical analysis undertaken herein- *See Results*), the following analysis was proposed:

- **Chi-square test of independence**: A Chi-square test was undertaken to ascertain if the asymmetry observed with the acetabulum was fluctuating, or directional (where one side shows the older phase more often than its counterpart). Such an analysis is vital for ascertaining the type of systematic approach that should be utilised while estimating age from the acetabulum (i.e., choosing one side over the other, or the older/younger acetabulum over its counterpart, or giving equal weightage to both sides).
- **Percentage of Directional Asymmetry (%DA)**: Percentage of directional asymmetry was computed to empirically demonstrate the extent of directionality within the acetabulum using the following Auerbach and Ruff’s formula [55]:

$$\%\mathrm{DA}=\left(\mathrm{Right}-\mathrm{Left}\right)/\left(\mathrm{Average}\;\mathrm{of}\;\mathrm{right}\;\mathrm{and}\;\mathrm{left}\right)\ast100$$

Within this formula, right and left refer to scores allotted to the right and left acetabulum, respectively, for individual acetabular variables. %DA values were thus computed for each acetabular variable separately, to comment on the extent of directionality/asymmetry within individual variables. Within this study, overall %DA values were not computed to circumvent masking the impact of asymmetry on individual variables, as the latter approach has the potential to influence the generation of weighted age estimation methods. For this statistical computation, to differentiate between slight side biases and true directionality, only individuals with %DA ≥ 0.5% were included. The total number of right biased and left biased individuals were then compared for equivalency (50:50) using the Chi-square test, following the method prescribed by Auerbach and Ruff [55].

iii) **Mean %DA values**: Mean %DA values were computed using the percentage of directional asymmetry for males and females separately, for each acetabular variable, by taking into consideration slight as well as significant side biases. Given the non-parametric nature of the data, median %DA values were also computed for the same.

The Chi square test, percentage of directional asymmetry and mean %DA values were computed for the two sexes individually, and for different age classes (10–39; 40–64; 65+) of both sexes separately. This was done to assess the prevalence of asymmetry and its directionality within both sexes, and with increasing age.

d) **Observer error and asymmetry**: Given the virtual aspect attached to this study, observer error (inter and intra) analysis was undertaken previously [19]. Inter- and intra-observer error was assessed using Cohen’s κ within this 2022 study [19]. Sixty samples (left acetabulum alone) were evaluated by two independent observers (lead investigator and another experienced forensic anthropologist) to obtain the inter-observer error. The same 60 samples were re-evaluated by the lead investigator (VW) after a duration of 3 weeks from the time of initial assessment to compute the intra-observer error. The obtained values were interpreted using the system described in Altman [56] which is as follows: κ < 0.20: poor agreement, κ = 0.21–0.40: fair agreement, κ = 0.41–0.60: moderate agreement, κ = 0.61–0.80: good agreement, κ = 0.81–1.00: very good agreement.

In order to negate/factor-in the contribution of observer errors towards the observed bilateral asymmetry, intra-observer scores were utilised herein. Only the left acetabulum was utilised for computing this estimation, following the principle of independence of data points recommended by Overbury et al. [2]. To assess whether the variability between pairs of acetabula arises due to observer error, or indeed due to asymmetry, the two variances (1st observation for the left acetabulum - 2nd observation for the left acetabulum; right acetabulum - left acetabulum) were compared. In addition to this, the Spearman correlation between observer error (1st observation for the left acetabulum - 2nd observation for the left acetabulum) and asymmetry (right acetabulum - left acetabulum) was also estimated, to establish associations, if any, between higher observer errors and incidence of asymmetry.

#### Impact of directional asymmetry on age estimation

Bilateral asymmetry and characteristic directionality within the acetabulum warrants establishing, and utilising the most methodologically and statistically sound approach for acetabular age estimation. A previous study by Warrier et al. [19] undertook acetabular age estimation through regression analysis and principal component analysis (PCA). The five variables analysed in this study were subjected to PCA and a weighted summary age model was created wherein weights were assigned to each variable in relation to the first principal component. Bayesian regression is another form of statistical analysis that may be utilised for age estimation. Unlike frequentist regression, Bayesian regression aims to find the distribution of parameter values by incorporating a prior population [57]. As an added advantage, Bayesian regression can circumvent the issues of age mimicry, a limitation associated with frequentist regression [58]. In 2019, Nikita and Nikitas [58] advocated for the use of regression models which incorporate informative priors to enable more representative age estimation with the auricular surface of the ilium. Based on their findings they concluded that these statistical methods are just as reliable as Bayesian analysis, given that the correct statistical models are used, and with appropriate training samples and/or priors. However, such an approach has never been applied to the acetabulum.

The present study employed the use of these Bayesian multiple regression models to compute error rates for acetabular age estimation. Due to sample size constraints, a uniform prior was utilised here in lieu of a specific informative prior. This approach will also allow for greater generalisability of this study’s findings [59]. A weighted approach was also incorporated through the use of Likelihood Ratios, wherein the weights were estimated using an inverse function (inverse of the variances for each predictor variable were approximated as the weight; as lesser variance indicates greater precision. Thus, more precise variables were assigned greater weight). The study sample was broken down into a training set comprising of 363 individuals, and a test set of 100 (50 males and 50 females) individuals using a random number generator. Bayesian regression models were fit and derived using the training set, and its predictive performance was assessed using the test set. Further complicated statistical analysis was not undertaken at this point, as establishing whether asymmetry does indeed have a significant impact on accuracy and reliability for age estimation, was the priority of this study. In case a significant impact is observed, it is worth further investigating the same using comprehensive Machine Learning models, which remain the most applicable and accurate statistical approach within Forensic Anthropology and Medicine [22, 60].

Predictive performance using the test set was computed as inaccuracy, bias, and root mean square error (RMSE) values. These error computations were established for males and females, and the combined population, overall, using the following formulae:

$$\mathrm{Inaccuracy}=\frac{\mathit\Sigma\mathit\:\mathit{\left(\left|Estimated\:age\:using\:Bayesian\:regression-Chronological\:age\right|\right)}}{\mathit n}$$

$$\:\mathrm{Bias}=\frac{\mathit\Sigma\mathit\:\mathit{\left({Estimated\:age\:using\:Bayesian\:regression-Chronological\:age}\right)}}{\mathit n}$$

$$\begin{array}{lc}\mathrm{RMSE}=\\\frac{\sqrt{\sum\:_{i=1}^n({Estimated\:age\:using\:Bayesian\:regression-Chronological\:age)}^2}}n\end{array}$$

All three error computations were computed using the codes created for Bayesian regression analysis in RStudio.

Furthermore, Bayesian regression analysis was undertaken using different approaches:

1. Inaccuracy, bias and RMSE values were computed using only either side of the acetabulum (left acetabulum only and right acetabulum only).
2. Inaccuracy, bias, and RMSE values were computed using only either side of the acetabulum (acetabulum with the older score always, and acetabulum with the younger score always).
3. Inaccuracy, bias, and RMSE values were computed using both sides of the acetabulum together (both right and left side scores were utilised; the scores were not averaged, instead weights were assigned to scores of both sides). This approach was utilised to establish if employing both sides garners more accurate and precise age estimates, as compared to using one side alone.

These multiple approaches were undertaken to establish the most accurate age estimation approach (i.e., choosing one side over the other, or consistently choosing the older/younger acetabulum for age estimation, or computing a value using both sides). The most accurate age estimation approach was established, using the predictive performance values, for the combined population, and for the two biological sexes, across all age groups.

Asymmetry analysis was undertaken using IBM SPSS (Statistical Package for Social Sciences) 29.0 and Bayesian regression analysis was performed with RStudio and R 4.4.3. For all computations, a p-value < 0.05 was considered statistically significant.

## Results

### Prevalence of directional asymmetry

Overall, no statistically significant sex differences (*p* > 0.05) were observed between the mean ages for ordinal scores allotted to the right, as well as left acetabulum using the Mann-Whitney U test, barring stage 3 of the acetabular rim shape, and stage 1 of the acetabular rim porosity for the right acetabulum (Table 3). Despite the lack of a significant sexual dimorphism with the scoring technique, all subsequent statistical analysis was undertaken for the two sexes individually, to assess the incidence, prevalence, and impact of asymmetry separately.

**Table 3:** Descriptive statistics associated with different stages for the five acetabular variables, separated by sex and side

Acetabular variable	Stage	Side	Males	Females	*p-*value
Acetabular groove	0	R	15.88 ± 3.834	26.80 ± 21.959	0.508
		L	15.88 ± 3.834	33.67 ± 28.024	0.305
	1	R	28.56 ± 9.437	33.97 ± 14.979	0.171
		L	28.65 ± 9.822	30.36 ± 12.957	0.939
	2	R	50.67 ± 12.626	50.38 ± 11.650	0.918
		L	50.02 ± 12.366	50.36 ± 11.221	0.927
	3	R	64.52 ± 10.765	62.88 ± 10.459	0.336
		L	64.02 ± 11.389	62.56 ± 10.897	0.373
Acetabular rim shape	0	R	21.50 ± 6.525	24.38 ± 11.913	1.000
		L	20.33 ± 7.078	24.23 ± 12.015	0.981
	1	R	24.31 ± 9.772	29.00 ± 13.678	0.261
		L	24.96 ± 9.504	29.17 ± 13.517	0.351
	2	R	29.67 ± 12.342	34.33 ± 23.288	1.000
		L	26.71 ± 9.673	34.33 ± 23.288	0.907
	3	R	30.11 ± 5.287	37.44 ± 9.993	**0.015**
		L	31.05 ± 6.911	34.79 ± 7.127	0.115
	4	R	46.44 ± 9.952	49.25 ± 9.357	0.110
		L	45.41 ± 10.852	47.48 ± 9.899	0.121
	5	R	56.13 ± 13.171	55.92 ± 12.171	0.973
		L	55.59 ± 13.706	56.76 ± 10.586	0.911
	6	R	65.48 ± 11.502	65.35 ± 10.569	0.890
		L	66.47 ± 9.490	65.21 ± 11.942	0.921
Acetabular rim porosity	0	R	22.43 ± 5.713	25.56 ± 11.325	0.273
		L	21.32 ± 6.301	27.26 ± 14.441	0.312
	1	R	29.92 ± 9.685	37.68 ± 14.368	**0.046**
		L	30.29 ± 10.081	36.75 ± 13.026	0.345
	2	R	43.34 ± 10.831	47.07 ± 11.103	0.375
		L	42.24 ± 10.635	47.02 ± 10.339	0.217
	3	R	50.63 ± 10.592	53.17 ± 12.920	0.972
		L	51.53 ± 10.522	52.57 ± 13.219	0.981
	4	R	65.59 ± 9.450	63.37 ± 10.060	0.891
		L	65.95 ± 9.528	62.57 ± 10.054	0.797
	5	R	64.50 ± 11.271	62.13 ± 10.837	0.921
		L	64.79 ± 11.237	63.50 ± 10.739	0.893
Apex activity	0	R	20.18 ± 6.645	18.71 ± 5.438	0.219
		L	17.88 ± 8.442	19.33 ± 5.132	0.371
	1	R	27.91 ± 9.479	32.69 ± 13.673	0.473
		L	25.72 ± 6.347	29.53 ± 12.648	0.569
	2	R	45.62 ± 13.376	48.46 ± 11.357	0.791
		L	44.58 ± 13.333	47.98 ± 11.689	0.891
	3	R	58.79 ± 12.162	58.55 ± 12.433	0.973
		L	57.62 ± 12.540	59.55 ± 11.033	0.761
	4	R	67.48 ± 11.199	71.25 ± 5.500	0.912
		L	67.67 ± 9.557	63.29 ± 10.029	0.579
Activity on the outer edge of the acetabular fossa	0	R	25.64 ± 8.856	31.07 ± 14.811	0.208
		L	26.31 ± 10.859	31.67 ± 16.179	0.275
	1	R	45.15 ± 14.176	48.71 ± 12.737	0.129
		L	44.83 ± 14.609	47.55 ± 12.810	0.213
	2	R	63.35 ± 11.465	59.84 ± 12.494	0.769
		L	62.81 ± 11.848	62.66 ± 9.597	0.891

p-values for sex differences in mean age values have been estimated using the Mann-Whitney U test. p-values in bold indicate statistically significant differences in mean ages between males and females

*R * Right acetabulum, *L * Left acetabulum

- Incidence of asymmetry in relation to biological sex

**Fig. 1:**
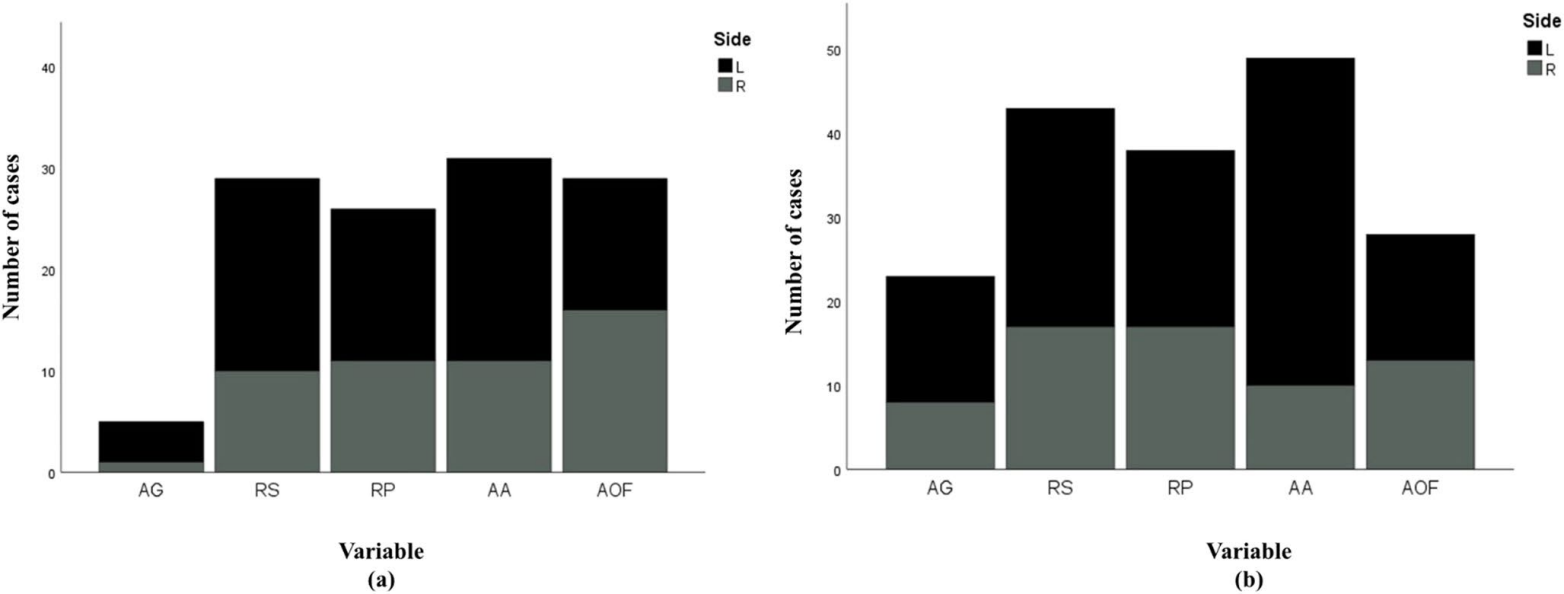
Directionality within acetabular variables indicated by the left side demonstrating the older score in a majority of cases. (**a**) Females; (**b**) Males; AG= Acetabular groove; RS= Rim shape; RP= Rim porosity; AA= Apex activity; AOF= Activity on the outer edge of the acetabular fossa; L= Left; R= Right. Black indicates the number of cases where the left side scored higher, and grey indicates number of cases where the right side scored higher. Stacked bars in this chart have been created using equivalency ratios listed in Table 4

Bilateral differences when evaluated separately for the two sexes indicated statistically significant differences only for the acetabular groove (*p* = 0.018) in females, and apex activity (*p* < 0.001) in males. In both sexes, the left side scored higher (older) than the right for all variables, except for activity on the outer edge of the acetabular fossa in females. The Chi square test of independence illustrated the presence of directional asymmetry within the specific bilaterally different variables, in both sexes (*p* < 0.001). Despite the lack of any statistically significant bilateral differences with the remaining four variables in males and females, these variables, too, demonstrated characteristic directionality (*p* < 0.001) (Fig. 1). Given this finding, percentage of directional asymmetry was computed for all five variables in both sexes.

Percentage of directional asymmetry yielded negative values , suggesting a left predominance for acetabular variables in both sexes. Mean values for percentage of directional asymmetry (Table 4), too, indicated a left predominance (negative values) for all five variables in males and females, with the exception of acetabular rim porosity in females. As both findings (%DA and mean %DA) garner the same interpretation, only mean %DA values have been listed here for context. It is important to mention here that %DA could not be computed for cases where both acetabula scored 0, yielding an average of 0. Such individuals/cases were manually excluded from the mean %DA calculations, and were automatically excluded during the test of equivalency.

**Table 4:** Mean %DA and equivalency of directionality within acetabular variables, separated by sex

Acetabular variable	Females		Males	
	Mean %DA (*N*)	Equivalency ratio^#^	Mean %DA (*N*)	Equivalency ratio^#^
Acetabular groove	-4.13* (192)	4:1	-1.17 (260)	15:8
Acetabular rim shape	-1.18 (183)	19:10	-4.85 (254)	26:17
Acetabular rim porosity	0.26 (180)	15:11	-6.36 (243)	21:17
Apex activity	-5.22 (192)	20:11	-10.04* (260)	39:10
Activity on the outer edge of the acetabular fossa	-0.38 (171)	13:16	-3.70 (223)	15:13

*DA* Directional asymmetry; #Ratio of individuals demonstrating left: right side dominance; *Variables with statistically significant bilateral differences; N = sample size; Mean %DA has been calculated using the computable sample size where average ≠ 0, whereas equivalency ratio (left: right) has been computed with %DA ≥ 0.50. Sample size associated with the Mean %DA calculation has been tabulated within brackets for statistical context. The same sample size was employed within equivalency ratio estimations as no samples displayed %DA values between 0–0.5.5

Subsequently, when %DA data was subjected to the test of equivalency (Table 4), non-equivalent numbers were obtained demonstrating population level lateralisation favouring the left acetabulum, for all five variables in males, and the first four variables in females. Despite the presence of statistically significant bilateral differences for certain variables, and a characteristic degree of lateralisation, median %DA values were 0.00 for all five variables in both sexes. A comparison of mean %DA values between the two sexes indicates that barring the acetabular groove, males yielded a greater extent of directionality (more negative mean %DA values) for acetabular variables. However, mean %DA values obtained for each variable in both sexes, when examined for sex differences using the Mann Whitney U test, yielded a *p* > 0.05 (*p* = 0.222), indicating an absence of sexual dimorphism with regards to extent and degree of directionality.

A crosstab analysis depicting degree of asymmetry (Table 5) demonstrated that the scores of the acetabular rim shape, and activity on the outer edge of the acetabular fossa differ by a magnitude of one score alone, in 14.87% females, whereas the acetabular groove differs by a magnitude of one score alone in 11.56% males. For all other variables in both sexes, scores differed by a magnitude of 1, as well 2 scores, the latter, marginal, in comparison to the former.

**Table 5:** Magnitude of asymmetry within different acetabular variables separated by sex

	Females		Males	
Acetabular variable	*N* ± 1	*N* ± 2	*N* ± 1	*N* ± 2
Acetabular groove	7.17%	0.51%	11.56%	-
Acetabular rim shape	14.87%	-	15.29%	0.74%
Acetabular rim porosity	12.09%	2.05%	12.68%	1.49%
Apex activity	15.38%	0.51%	17.91%	0.37%
Activity on the outer edge of the acetabular fossa	14.87%	-	9.70%	0.37%

Percentages have been calculated in proportion to total sample sizes for the two sexes for each variable; *N* ± 1 = A difference of one score; *N* ± 2 = a difference of two scores; dashes indicate the absence of any differences in scores of the right and left acetabulum for the specific category

b) Incidence of asymmetry in relation to chronological age

In order to comment on the incidence of asymmetry and its relationship with age, the study set was divided into three age classes (10–39 years, 40–64 years, and 65 + years) (Table 2). Bilateral differences when evaluated for different age groups in females did not indicate any statistically significant differences (Table 6). Bilateral differences when evaluated for different age groups in males, indicated statistically significant differences in rim shape (*p* = 0.007), rim porosity (*p* = 0.014) apex activity (*p* = 0.001), and activity on outer edge of the acetabular fossa (*p* = 0.034) in ≤ 39 year old males, and apex activity (*p* = 0.012) in 65 + year males (Table 7).

**Table 6:** Mean %DA, median %DA, and equivalency of directionality within acetabular variables, separated by age in females

	Computed statistic			
Acetabular variable	Mean/median %DA (*N*)	Equivalency ratio^#^	Age group (years)	*p* -value**
Acetabular groove	-88.88/-66.66 (05)	4:1	10–39	0.180
	-24.76/-40.00 (07)	5:2	40–64	0.257
	-60.00/-40.00 (03)	3:0	65+	0.102
Acetabular rim shape	7.40/22.22 (03)	1:2	10–39	0.564
	-9.72/-22.22 (20)	7:3	40–64	0.074
	-7.40/-18.18 (06)	2:1	65+	0.414
Acetabular rim porosity	-8.00/-40.00 (05)	3:2	10–39	1.000
	2.19/22.22 (13)	6:7	40–64	0.971
	7.30/-25.39 (08)	3:1	65+	0.157
Apex activity	-86.66/-133.33 (08)	5:3	10–39	0.564
	-18.26/-40.00 (17)	12:5	40–64	0.074
	0.00/0.00 (06)	1:1	65+	1.000
Activity on the outer edge of the acetabular fossa	-22.22/66.66 (09)	4:5	10–39	0.739
	-5.12/66.66 (13)	6:7	40–64	0.782
	28.57/66.66 (07)	3:4	65+	0.705

*DA* Directional asymmetry; #Ratio of individuals demonstrating left: right side dominance; No variables with statistically significant bilateral differences were seen for this sex; Mean %DA and equivalency ratios have been calculated using the computable sample size wherein average ≠ 0 and %DA ≥ 0.50, respectively. Sample size associated with the Mean %DA calculation has been tabulated within brackets for statistical context. The same sample size was utilised for equivalency ratios as no samples displayed %DA values between 0–0.5.5

** p-values correspond to Wilcoxon test undertaken to establish bilateral asymmetry for different acetabular variables in different age classes

**Table 7:** Mean %DA, median %DA, and equivalency of directionality within acetabular variables, separated by age for males

	Computed statistic			
Acetabular variable	Mean/median %DA (*N*)	Equivalency ratio^#^	Age group (years)	*p* -value**
Acetabular groove	-13.33/-66.66 (05)	3:2	10–39	0.655
	-10.90/-40.00 (11)	7:4	40–64	0.366
	-17.14/-40.00 (07)	5:2	65+	0.257
Acetabular rim shape	-66.23/-47.61 (20)	4:1*	10–39	**0.007**
	7.53/22.22 (12)	5:7	40–64	0.317
	0.03/18.18 (11)	5:6	65+	0.782
Acetabular rim porosity	-103.73/-200.00 (13)	7:2*	10–39	**0.014**
	25.55/40.00 (11)	3:8	40–64	0.090
	4.32/22.22 (09)	4:5	65+	0.739
Apex activity	-140.95/-200.00 (14)	13:1*	10–39	**0.001**
	-11.57/-40.00 (19)	13:6	40–64	0.059
	-26.19/-40.00 (16)	13:3*	65+	**0.012**
Activity on the outer edge of the acetabular fossa	-150.00/-200.00 (08)	7:1*	10–39	**0.034**
	4.44/40.00 (09)	4:5	40–64	0.255
	30.30/66.66 (11)	4:7	65+	1.000

*DA* Directional asymmetry; #Ratio of individuals demonstrating left: right side dominance; *Variables with statistically significant bilateral differences; Mean %DA and equivalency ratios have been calculated using the computable sample size wherein average ≠ 0 and %DA ≥ 0.50, respectively. Sample size associated with the Mean %DA calculation has been tabulated within brackets for statistical context. The same sample size was utilised for equivalency ratios as no samples displayed %DA values between 0–0.5.5

** p-values correspond to Wilcoxon test undertaken to establish bilateral asymmetry for different acetabular variables in different age classes. p-values in bold indicate statistically significant differences in mean ages between males and females.

The Chi square test of independence demonstrated the presence of directionality with all five variables across all age groups in both sexes (*p* < 0.001). In majority of the cases, there was a greater proportion of higher left: right ratio, as compared to higher right: left ratio in females (Table 6), and males (Table 7), as indicated by equivalency ratios. This suggests a largely left lateralisation. This is further corroborated by negative mean %DA values. However, there were also certain exceptions to this dictum; acetabular rim shape and activity on the outer edge of the acetabular fossa for ≤ 39 year old females, acetabular rim porosity and activity on the outer edge of the acetabular fossa for 40–64 year old females, apex activity and activity on the outer edge of the acetabular fossa in 65 + females (Table 6). Similarly, in males (Table 7), acetabular rim shape, rim porosity, and activity on the outer edge of the acetabular fossa in the 40–64 and 65 + years age categories garnered a greater indication of right lateralisation with higher right: left equivalency ratios, and positive mean and median %DA values.

Amongst all five variables, acetabular groove garnered highest mean %DA values, and apex activity yielded highest median %DA values for females of the ≤ 39 years age category. For the 40–64 years and 65 + age classes, highest mean %DA value was observed with acetabular groove, and highest median %DA was obtained with activity on the outer edge of the acetabular fossa (Table 6). For males of the young and old age classes, highest mean and median %DA values were computed with the activity on the outer edge of the acetabular fossa. For the middle age class, acetabular rim porosity garnered highest mean and median %DA values (Table 7). A Kruskal-Wallis test demonstrated the lack of statistically significant differences in mean and median %DA values between different age classes in males as well as females (*p* > 0.05).

Table 8 tabulates the degree of asymmetry for different variables across different age cohorts for both sexes. All five variables demonstrated asymmetry by a factor of one score (*N* ± 1) in a greater proportion of cases. *N* ± 2 degree of asymmetry was largely exhibited in middle and old age classes of females and males, and with the acetabular rim porosity in younger males.

**Table 8:** Magnitude of asymmetry within different acetabular variables, separated by age classes and sex

Age class (years)	Acetabular variable	Females		Males		
		*N* ± 1	*N* ± 2	*N* ± 1	*N* ± 2	
10–39	Acetabular groove	10.63%	-	5.20%		-
40–64		6.36%	-	12.08%		-
65+		7.89%	-	8.64%		-
10–39	Acetabular rim shape	6.38%	-	20.83%		-
40–64		18.18%	-	12.08%		1.09%
65+		15.78%	-	12.34%		1.23%
10–39	Acetabular rim porosity	8.51%	-	16.66%		5.20%
40–64		9.09%	2.72%	10.98%		1.09%
65+		21.05%	-	9.87%		1.23%
10–39	Apex activity	17.02%	-	14.58%		-
40–64		14.54%	0.90%	19.78%		1.09%
65+		15.78%	-	19.75%		-
10–39	Activity on the outer edge of the acetabular fossa	19.14%	-	8.33%		-
40–64		11.81%	-	9.89%		-
65+		18.42%	-	12.34%		1.23%

Percentages have been calculated in proportion to sample sizes for each age class for each variable; *N* ± 1 = A difference of one score; *N* ± 2 = a difference of two scores; dashes indicate the absence of bilateral asymmetry for the specific category

c) Observer error and asymmetry

Inter and intra-observer errors were shown to lie in the range of moderate to very good agreement within and between observers [19]. From the examined pairs for the observer error, 6–10% pairs showed different scores, as opposed to the 6–18% pairs which showed asymmetric differences. Additionally, the variance due to intra-observer error pairs (0.12–0.98) was slightly lower than the variance associated with asymmetric pairs (0.16–0.98), indicating that asymmetry cannot be attributed to observer error alone. However, the slight overlap in variance also suggests that some asymmetry can be attributed to observer subjectivity. Correlation values between observer error and asymmetry ranged from -0.667 to -0.795, suggesting that asymmetric pairs do not necessarily show higher observer error, and vice versa.

### Impact of directional asymmetry on age estimation

Table 9 shows a comparison of the error rates in females, males and the overall combined population, considering all age categories pooled together. In females, results indicate that the lowest inaccuracy and RMSE values were obtained with the right acetabulum, and the lowest bias was obtained with the younger acetabulum (Table 9). In males, results indicated that the lowest inaccuracy, bias and RMSE values were obtained with the right acetabulum (Table 9). In the overall combined population, results indicated that the lowest inaccuracy, bias, and RMSE values are obtained when the right acetabulum alone is employed for age estimation (Table 9).

**Table 9:** Error values obtained with different acetabular age Estimation approaches, separated by biological sex

Sex	Acetabular age estimation approach	Inaccuracy (years)	Bias (years)	RMSE (years)
Females	Left acetabulum only	14.72	11.85	16.67
	Right acetabulum only	7.58	-1.22	10.88
	Older acetabulum only	13.23	10.01	15.34
	Younger acetabulum only	7.66	-0.43	10.99
	Both acetabula	18.54	16.28	20.34
Males	Left acetabulum only	14.02	12.61	16.36
	Right acetabulum only	7.19	0.86	9.86
	Older acetabulum only	13.48	11.92	15.86
	Younger acetabulum only	15.91	-15.25	18.19
	Both acetabula	7.76	2.39	10.15
Combined population	Left acetabulum only	14.46	12.30	16.66
	Right acetabulum only	7.50	-0.33	10.53
	Older acetabulum only	13.41	10.98	15.63
	Younger acetabulum only	7.64	0.49	10.59
	Both acetabula	10.39	6.93	12.68

*RMSE* Root mean square error; older acetabulum signifies the acetabulum which scored higher between the two halves of the pelvis; younger acetabulum signified the acetabulum which scored lower between the two halves of the pelvis

## Discussion

The previous two decades have seen the conception, followed by an incremental rise, in the use of the acetabulum for age estimation [23]. The acetabulum confers numerous advantages to age estimation and human identification, attributable to its highly durable and resistant nature [17], anatomical construct and positioning [61]. This also increases the probability of finding this marker within investigative contexts. Furthermore, a meta-analysis published in 2023 [23] demonstrated the higher accuracy and reliability associated with the acetabulum for age estimation, when compared to other commonly utilised markers. Presently, there is no published literature examining the prevalence of asymmetry within the right and left acetabulum and its impact on the accuracy of age estimation and human identification, despite the presence asymmetry having been demonstrated before. The present study, was thus, designed to explore this asymmetry in-depth, and evaluate the implications of the same.

### Incidence of asymmetry in relation to biological sex

Bilateral differences analysis indicated statistically significant differences for specific acetabular variables in both sexes, and the percentage of directional asymmetry, mean %DA values, and equivalency ratios pointed towards a characteristic left lateralisation (i.e., directionality and/or directional asymmetry) for all acetabular variables in males, and most variables in females. Interestingly, despite the left predominance observed with the acetabular rim porosity in females, the mean %DA value for this variable was overall, positive, albeit quite close to a zero. Furthermore, with activity on the outer edge of the acetabular fossa in females, comparable lateralisation values were obtained, with the right acetabulum outscoring its counterpart by a proportion of three. This indicates possible fluctuating asymmetry with rim porosity and activity of the outer edge of the acetabular fossa in females alone.

Similar instances of interspersed asymmetry have been reported previously [46]; however, this was with males of European and American populations. Campanacho in this 2016 study, reported that for European and American collections, the right side, largely, scored higher/appeared older, whereas males of the American collection alone, yielded left side dominance in the acetabular groove [46].

Asymmetry within the acetabulum is to be expected given the disproportionate mechanical stresses endured by the pelvic joint and the lower environmental plasticity of these articulating regions. This is particularly true for the femoral head, due to idiosyncratic preferred limb dominance with regards to propulsion and kinetic loading response [31]. Furthermore, the vicinity of the acetabulum to the hind limbs forms the basis for the plausible hypothesis that this marker would mirror asymmetry exhibited by the femur [2, 29]. Previous investigations with the femur and lower limb have indicated a similar left dominance [62–67], which has been attributed to differences in kinematic variables and asymmetry in the contraction of certain limb muscles [31].

The predominance of left lateralisation, by and large, and thus accelerated aging with this side, supports the concept of crossed symmetry within the human body [65]. With rim porosity and activity on the outer edge of the acetabulum, the presence of fluctuating asymmetry demonstrates comparable paces of aging within both acetabula, in females. However, even with these two variables, based on the comparable equivalency ratios obtained here, the left acetabulum cannot be stated to have a significantly slower aging compared to its counterpart. This acceleration in aging within the left side of the human skeletal framework has been previously reported with other non-acetabular markers such as the epiphyseal union within limb bones, the iliac crest and ischial tuberosity, pelvic canal and non-canal variables, lower limb measures of maximum length, epicondylar breadth, hand and wrist bones, and the iliac auricular surface [26, 29, 55, 66, 68–70], and with certain acetabular variables such as angles of superior lunate surface of the acetabulum [71]. The differences in the degree of lateralisation across these five variables could be attributed to the differential wear and tear within the acetabulum. This facet of different skeletal features exhibiting varying levels of asymmetry in response to the same mechanical environment has been posited previously by Auerbach and Ruff [55]. An in-depth anatomical investigation into biomechanical loading, wear and tear due to occupations and lifestyle, and femoral mirroring within the acetabulum can further help rationalize these observed differences. A systematic investigation into different lived history experiences, and factors capable of affecting the variability of anatomical expression within acetabular variables might also help shed light on these differential findings between the two sexes.

Mean %DA values when compared between the two sexes, indicated the absence of any significant sexual dimorphism, corroborating previous findings with the lower limbs and certain pelvic variables [29, 55, 72, 73]. It is important to mention here that amongst the pelvic traits examined within these studies, acetabular features were not evaluated, with the closest mirroring dimension being the femoral head diameter. This finding, along with the lack of sex differences in mean ages for majority ordinal scores suggest that accurate age estimation can be undertaken successfully even when biological sex cannot be established with certainty within this population (for e.g. in case of fragmented remains). This finding contradicts previous studies which reported significant sex differences in acetabular aging rates [36, 41], suggesting population level differences. It is interesting to note that in the study by San-Millán et al., [41] more stages displayed sexual symmetry (55.9%), as opposed to sexual dimorphism, indicating that sexual dimorphism across these variables cannot be generalised.

While mean %DA values did not differ between the two sexes, males, by and large, illustrated more negative mean values. The slightly greater degree of directionality in males (more negative mean %DA values), irrespective of the presence of asymmetry or not, is worth exploring. It has been proposed previously that conflicting selective pressures of bipedalism and obstetrics limit expression of variability in females [74, 75]. However, little information is available about the obstetric history of this study sample, and several other factors are believed to influence the variability expressed by the human pelvis [72, 76]. This constitutes another facet of asymmetry related research that should be investigated in the future. It is also interesting to note that previously a higher frequency of asymmetry in males has been reported with certain upper and lower limb dimensions [55, 68, 77]. Acetabular groove alone displayed more negative mean %DA values in females. While this can be attributed to the asymmetry observed with this variable in females and its lack thereof in males, this finding requires further validation through additional research endeavours. The present study utilised an unequal representation of the two biological sexes, and findings described above should be further corroborated through the use of balanced study sets.

An analysis of the degree of asymmetry indicated that the major proportion of asymmetric cases (irrespective of its statistical significance) fall into the ± 1 asymmetry category. Thus, the degree of asymmetry was rarely drastic, and is most likely the result of biomechanical factors, or a transition between adjacent stages, as opposed to pathological conditions [78]. However, the present study cannot empirically corroborate this as the sample selection procedure involved excluding individuals suffering from severe degenerative and pathological diseases [19].

### Incidence of asymmetry in relation to chronological age

The impact of age on acetabular asymmetry was investigated within this study, with certain noteworthy findings.

Specific variables within different age categories in both sexes displayed bilateral asymmetry. By and large, the left acetabulum demonstrated the older score (and negative mean %DA values) in different age categories in both sexes, corroborating previously reported findings [26, 29, 55, 66, 68, 71]. It is interesting to note that for activity on the outer edge of the acetabular fossa for the young and middle aged females, even though equivalency ratios indicated right dominance, overall negative mean %DA values and overall positive median %DA values were obtained. Similarly, acetabular rim porosity in older females yielded positive mean %DA values, but negative median %DA values and left lateralisation equivalency ratios. Both these findings corroborate the previous hypothesis of fluctuating asymmetry within these variables in this sex.

Based only on the mean and median %DA values across different age categories in females, degree of directionality appeared to initial decrease with age and then increase again for the acetabular groove, rim porosity, apex activity, and activity on the outer edge of the acetabular fossa. For rim shape, the reverse was observed with degree of directionality initially increasing with age and then decreasing for the 65 + age class. In males, a similar trend of initially decreasing and then increasing directionality with age was observed with the acetabular groove, apex activity and activity on the outer edge of the acetabular fossa. For rim shape and rim porosity, a trend of decreasing directionality with age was observed. While the degree of directionality has not been investigated before, past research has identified patterns of increasing asymmetry with age in females, and decreasing asymmetry with age in male primates [26, 79]. These findings corroborate the theory of a non-uniform progression of physiological changes with age, put forth by Overbury et al. [2].

It has been conjectured that when areas of the skeleton experience greater biomechanic stress, as is common in younger individuals, they may grow and mature at a faster rate or at an earlier age [26]. Subject to the extent and action of these biomechanic stressors, an asymmetric maturation might ensue within this age group. Similarly, it has been hypothesized that bilateral asymmetry in older ages could be due to environmental stresses as well as biomechanic stresses [26]. Both these arguments could build into the unimodal and bimodal directionality patterns observed herein, and requires further investigation. The present study employed an unequal representation of individuals across the three age classes, and similar investigations utilising balanced age groups can help rule out the effect of a non-homogenous study set on observed patterns.

An analysis into the degree of asymmetry demonstrated that most *N* ± 2 asymmetries were restricted to individuals older than 40 years, suggesting accentuated magnification of physiological changes within the acetabulum with increasing age, resulting in a greater degree of asymmetry. This is also in accordance with the well-established Trajectory Effect [80] which states that the variability observed in the different age indicators increases with age and continues to increase throughout life.

Irrespective of the directionality of lateralisation, or the fluctuating asymmetry observed, the presence of bilateral differences warrants a careful application of acetabular age estimation models. The present study undertook acetabular age estimation using multiple approaches, to establish best practice when paired acetabula, regardless of their asymmetric nature, are available for examination.

### Impact of asymmetry on age Estimation

Across both sexes and the combined population, the right and/or younger acetabulum appeared to yield the lowest error computations for age estimation, irrespective of the nature of asymmetry, i.e., directional or fluctuating. Error values were computed separately for the total population here as well, in order to evaluate the impact of asymmetry on age estimation in cases where sex of the remains cannot be established with certainty.

The findings of the present study contradict previous practices of selectively utilising the left acetabulum for age estimation, whilst following standardised practices, as opposed to considering the counterpart, or both acetabula [41, 42]. This study also contradicts previous findings of the older side of the pelvis garnering more accurate estimates of age [2, 24]. However, these previous studies utilised the pubic symphysis, a marker which is significantly affected by lived experiences of individuals [81–83]. The acetabulum, on the other hand, has been shown to be relatively resistant to certain facets of an individual’s lived history [44]. Mays, in 2012, noted that individuals engaged in non-manual/non-exertive occupations exhibited older acetabular scores than those involved in manual labour, suggesting that physical activity might be acting as an inhibitor to acetabular aging [36]. Given that most people are right-handed, it is plausible that the right lower limb experiences more wear and tear. Thus, the right acetabulum experiences more biomechanical activity, potentially slowing age-related changes and resulting in lower age estimates and lower error computations. Given that in most cases, the younger scoring acetabulum was also the right acetabulum, this theory holds true for the lower error observed with the younger acetabulum.

Furthermore, the concept of crossed symmetry suggests that the left lower limbs will demonstrate older ages, and thus the right counterpart might be more representative of the true age. It is also important to point out that although acetabular aging methods involve an ordinal scoring, acetabular development and the subsequent appearance of age-related physiological changes do not necessarily follow a uniform path across individuals. Not all individuals go through every stage of the process. Aging may be discontinuous and/or non-linear, as it can be influenced by increasing biological variability [80] and by factors other than age, such as pathological conditions, nutrition, lifestyle, medical treatments, population ancestry, or individual genetic background, among others. Hence some bilateral differences are feasible given that changes will not happen at a fixed point in time across all individuals. Given this, it is vital to establish which side might yield more accurate results within the investigative context, instead of selectively utilising a singular side each time, as done previously by employing the left side.

The lack of any majorly statistically significant sex differences in mean ages for stages and degree of asymmetry indicates that acetabular age estimation using the SanMillán-Rissech method [41] can be successfully applied to remains to this population even when sex is not discernible. Furthermore, the lack of any statistically significant differences in the degree of asymmetry across different age classes also suggests that the applicability of this method will not be impacted by the varying expressions of asymmetry within different age categories or classes. Nevertheless, best practice dictates that age estimation for case resolution should be undertaken after a preliminary attempt to establish sex of the remains presenting for examination, wherever feasible, in order to obtain most precise results. This is particularly vital, given that specific acetabular variables evaluated in the present study demonstrated asymmetry in both sexes, and weighted age estimation approaches such the one utilised herein, might be impacted by these expressions of asymmetry. Age estimation when undertaken using individual variables alone, such as in Warrier et al. [19], will also provide more accurate results when observed asymmetry in sexes is appropriately accounted for. It is also recommended to not selectively utilise a single pre-determined acetabulum for age estimation, as the present study has demonstrated that for an Indian population, the right/younger acetabulum garners more accurate results, when compared to the commonly advocated left acetabulum. It is plausible that similar findings will be observed with other populations, and this should be further tested in future investigations.

Error computations obtained herein with the right acetabulum using Bayesian regression were comparable to those obtained with Principal Component Analysis within the original study [19], advocating for the use of weighted age estimation approaches. While the original study assigned weights using principal components, herein weights were assigned based on their variance through the use of likelihood ratios, following a more statistically robust approach. However, error computations obtained with the left acetabulum using Bayesian regression were higher than those obtained with Principal Component Analysis, attributable to the more comprehensive weighted variance approach utilised herein. However, in both studies, the right acetabulum garnered more accurate age estimates, indicating that the right acetabulum is more reliable for aging an Indian population. Similar investigations should be undertaken using this approach in different populations to further validate these findings.

### Observer error and asymmetry

A comparison of observer error and asymmetry did not reveal a significant impact of the former on the latter. In the previous study [19], there were no drastic differences observed in the analysis of the two independent observers, and it is plausible that the observations of a second observer, too, will not impact observed asymmetry, significantly. While the present study has effectively ruled out the role of intra-observer error on perceived asymmetry, future investigations should attempt to do so using multiple observers to corroborate this finding.

### Future research and recommendations

The present study has highlighted several unexplored facets of acetabular age estimation. Future investigations will attempt to further build up on these findings by exploring the impact of different environmental disturbances, genetic predispositions, and lived experiences on bilateral asymmetry within the acetabulum. Such factors are likely believed to the cause of disruptions within the developmental homeostasis, resulting in the observed asymmetry [26, 77, 84–86]. Furthermore, in agreement with Lazenby [87], magnitude, directionality, and impact of asymmetry is characteristic to populations, and should not be generalised for every population under scrutiny. Future investigations should attempt to assess the degree of asymmetry within different populations, to create a centralised, globalised approach towards acetabular age estimation, if possible. Similar investigations should also attempt to establish best practice when paired acetabula, regardless of their asymmetric nature, are available for examination, for different populations.

## Conclusion

The durability and high accuracy associated with the acetabulum renders it an age marker of significance within the context of human identification. The present study explored this prevalence of acetabular asymmetry, and its impact on age estimation. Statistically significant bilateral differences were observed within specific acetabular variables, with directional asymmetry in most acetabular variables, supported by a range of statistical tests. A greater degree of directionality, regardless of the presence or absence of asymmetry, was observed in males, with the non-homogeneity in the scoring of the two acetabula, appearing to increase with age in both sexes, with slightly erratic patterns observed within certain age classes. Regardless of the expression of asymmetry, or lack thereof, within the acetabulum, accurate estimates of age were obtained with the right/younger acetabulum in an Indian study sample, contradicting the previous systematic utilisation of the left acetabulum. The likelihood of the right/younger acetabulum garnering more accurate estimates of age, in comparison to the left, as shown in this study, should be further validated. An in-depth investigation into the anatomical constitution of the acetabulum, and the effects of ontogenic, genetic, pathological, environmental, lived experiences, and obstetric factors could additionally help rationalise the findings observed here.

## Data Availability

not applicable.
